# Correlation Between Chronic Tinnitus Distress and Symptoms of Depression: A Systematic Review

**DOI:** 10.3389/fneur.2022.870433

**Published:** 2022-05-02

**Authors:** Sebastiaan M. Meijers, Maaike Rademaker, Rutger L. Meijers, Inge Stegeman, Adriana L. Smit

**Affiliations:** ^1^University Medical Center Utrecht, Utrecht, Netherlands; ^2^Brain Center Rudolf Magnus, University Medical Center Utrecht, Utrecht, Netherlands; ^3^Radboud University Nijmegen Medical Centre, Nijmegen, Netherlands

**Keywords:** tinnitus, burden, depression, depressive symptoms, impact

## Abstract

**Objectives:**

In this systematic review, we aim to evaluate the evidence regarding the correlation between tinnitus distress and the severity of depressive symptoms in patients with chronic tinnitus. Also, the prevalence of clinically relevant depressive symptoms scores in patients with chronic tinnitus was evaluated.

**Methods:**

We performed a systematic review in PubMed, EMBASE, and the Cochrane library in June 2021 using the terms “depression” and “tinnitus,” and their synonyms, following PRISMA guidelines. Studies were selected on relevance and critically appraised regarding risk of bias using the Newcastle–Ottowa Quality Assessment Scale.

**Results:**

A total of 1,912 articles were screened on title and abstract after the removal of the duplicates. Eventually, 33 (1.5%) articles were included for the final analysis. Only cross-sectional cohort studies and case–control studies with a low level of evidence and a high risk of bias due to the study design and patient selection were found. Statistically significant correlations between the experienced tinnitus distress and depressive symptoms were reported in 31 out of 33 studies. Clinically relevant depression scores had a prevalence of 4.6–41.7%.

**Conclusions:**

In this systematic review, in which mostly cross-sectional studies were included, a statistically significant correlation was found between the experienced tinnitus distress and the reported severity of symptoms of depression in patients with chronic tinnitus. A wide range of clinically relevant depression scores were reported in included studies. Due to the high risk of bias of included studies it is not possible to provide a definite answer on the existence of this relationship. Future population-based studies are necessary to provide more clarity.

## Introduction

The subjective tinnitus is the perception of a noise or sound without an external acoustic stimulus (1). It is a phenomenon with a reported prevalence between 5.1 and 42.7% (2). The impact chronic tinnitus has on the patients differs widely and is associated with comorbidities such as sleep and/or concentration problems (3). Many patients with tinnitus have some kind of hearing impairment and their hearing status have been found to be correlated to the experienced tinnitus distress in previous studies (4, 5). As tinnitus itself is a symptom, the treatment options depend on the etiology of the underlying disease (6). However, in the majority of patients with tinnitus the underlying cause is unknown and symptom reduction seems to be the highest achievable goal for many patients affected. All in all, tinnitus can be responsible for a reduced quality of life and impaired psychological functioning (7).

The depressive feelings and depression have frequently been reported in patients with chronic tinnitus, with studies dating back till the 1980s (8). In 2019, Salazar et al. (9) published a review of the literature on the prevalence of depression in the adult populations with tinnitus, including patients with both chronic and acute tinnitus. From the 28 studies that were included, the authors distracted a mean prevalence of depression of 33% (range: 6–84%) (9). However, no details were provided about the experienced tinnitus distress, severity or impact on daily life in relation to the depression or depressive symptoms of the studied patients. In addition, several of the included studies did not use clear criteria on which the diagnosis depression was made. This is of importance for the interpretation of outcomes as in clinical practice the diagnosis depression can only be made by a physician and based on the Diagnostical and Statistical Manual of Mental disorders (DSM-V) (10), while depressive symptoms can be measured using a variety of questionnaires; most of which are self-rating questionnaires (11, 12).

In the earlier reports, several hypotheses have been made about the simultaneous finding of these entities. It is hypothesized that the impact of tinnitus on daily life can trigger a depression in depression-prone persons (9, 13, 14). In other studies, it was suggested that tinnitus-related stress can cause alterations in the limbic–cortical pathways of the brain which result in a more depressive state, similar to chronic pain (15–17). Although, so far, it is unknown if the relation between both is causal in one or the other way, or if the co-occurrence of both is related to other entities or variables.

More knowledge about the relationship between the experienced tinnitus distress and the severity of the depressive symptoms or the existence of a clinical depression in patients with chronic tinnitus is of upmost importance. More insight in this relationship would provide essential information for healthcare professionals in tinnitus care. Awareness of depressive symptoms or the existence of a depression could warrant specialized treatment for these patients aimed at relief for these symptoms apart from their tinnitus. Therefore, we aim to systematically asses the correlation between the severity of tinnitus distress and the severity of depressive symptoms. As a secondary objective, we aim to investigate the prevalence of clinically relevant depressive symptoms scores in patients with chronic tinnitus.

## Methods

We used the Preferred Reporting Items for Systematic Reviews and Meta-Analyses (PRISMA) guideline for this systematic review (18).

### Search Strategy

We conducted a systematic search of the literature on 26 June 2021. The electronic databases of PubMed, EMBASE, and the Cochrane Library were searched (Appendix 1). We used search terms and their synonyms of tinnitus and depressive symptoms or depression in title/abstract, medical subject headings (MeSH) terms, and Emtree fields. In addition to electronic database searches, reference lists were scanned to identify additional studies.

### Study Selection

**Figure 1:**
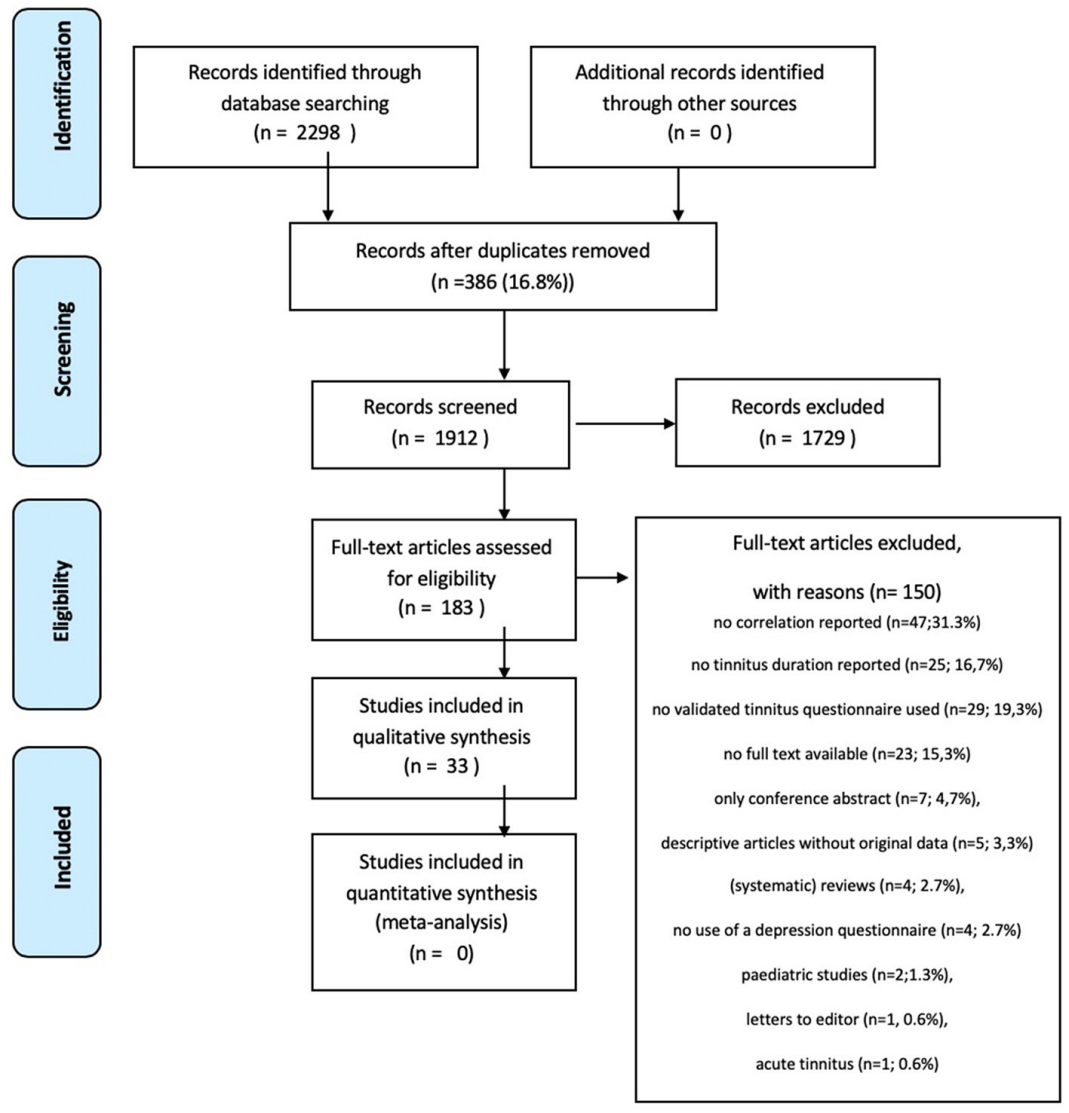
PRISMA 2009 flow diagram.

Three authors (SM, RM, and MR) independently scanned the initial search results on title/abstract to identify the studies, which met the predefined inclusion criteria. The retrieved studies were reviewed full text using the predefined inclusion and exclusion criteria (Figure 1). If an article was not available in full text, the author was contacted to request the full text article. Differences in opinion regarding the inclusion of studies were resolved by discussions.

We included the studies that described adults with chronic subjective tinnitus (defined as a minimum duration of 3 months). The randomized controlled trials and (retrospective and prospective) cohort studies reporting on the experienced tinnitus distress and depressive symptoms using validated questionnaires were selected. We excluded the pediatric studies, non-original study designs, animal studies, case reports (*n* <5), studies on the patients with cochlear implantation(s), studies with the overlapping populations or studies with non-available abstract or full text.

### Data Collection and Analysis

#### Quality Assessment of the Studies

Two authors (SM and MR) independently assessed the risk of bias of the included studies. The Newcastle–Ottawa Quality assessment scale (NOS) was used to assess the representativeness of the exposed cohort, selection of the non-exposed cohort (if present), ascertainment of exposure, demonstration that outcome of interest was not present at start of study (if possible), comparability (if possible), follow-up long enough for outcomes to occur, and adequacy of follow-up of cohorts. Two versions of NOS were used: one version for the cohort studies and one for the case–control studies. Using the NOS tool, the studies were judged to be of poor, fair or good quality (19). The scoring system is described in the legends of Supplementary Tables 1A,B, and they are provided as supplementary files.

#### Data Extraction and Synthesis

The following data was extracted by one author (SM): (1) The total number of participants in one study and their sex and age; (2) The type of tinnitus questionnaire (TQ) used; (3) The mean score on TQ; (4) The type of depression questionnaire used; (5) The mean score on depression questionnaire; (6) The clinical depression score (when reported); (7) The data about the correlation between tinnitus distress and depressive symptoms; (8) The data about hearing status of included participants. The second author (MR) checked the extracted data. The differences were solved in a consensus meeting.

#### Outcome Measures

The studies using multi-item validated questionnaires to measure tinnitus distress, severity or impact on daily life were included (Table 1A). These include the Tinnitus Handicap Inventory (THI), the TQ, the mini Tinnitus Questionnaire (mTQ), the Tinnitus Functional index (TFI), the Tinnitus Handicap Questionnaire (THQ), the Tinnitus Primary Function Questionnaire (TPFQ), and the Tinnitus Reaction Questionnaire (TRQ) (20, 21, 23–26, 28).

**Table 1A:** Characteristics of tinnitus questionnaires (TQ).

**Instrument**	**Developed to measure**	**Items**	**Scoring per item**	**Interpretation of score/grading system**	**Cronbach's α**	**Subscales**
THI (20)	Level of perceived tinnitus severity	25	0 never 2 sometimes 4 yes	0-16 light 18-36 mild 58-76 severe 78-100 catastrophic	0.93 (20)	Functional Emotional Catastrophic
TQ (21)	Psychological aspects of tinnitus complaints and distress	52^*^	0 not true 1 Partly true 2 true	0-30 light 31-46 mild 47-59 severe 60-84 very severe <46 = compensated >46 = decompensated	0.95 (22)	Emotional distress Cognitive distress Intrusiveness Auditory perceptual difficulties Sleep disturbance Somatic complaints
mTQ (23)	Abbreviated version of TQ	10	0 not true 1 Partly true 2 true	Grading system not provided. A higher total score indicates a higher level of tinnitus handicap	0.87 (22)	none
TRQ (24)	Psychological distress due to tinnitus	26	0 not at all 4 almost all the time	Grading system not provided. A higher total score indicates a higher level of tinnitus handicap	0.96 (24)	General distress interference Severity Avoidance
THQ (25)	The perceived degree of handicap due to tinnitus	27	0 strongly disagree 100 strongly agree	Grading system not provided. A higher total score indicates a higher level of tinnitus handicap	0.93 (25)	Physical health Emotional status Social consequences Hearing and communication Personal viewpoint
TFI (26)	Tinnitus severity and treatment related change	25	0 not affected 10 always affected	Grading system not provided. A higher total score indicates a higher level of tinnitus handicap	>0.7 (27)	Intrusiveness Sense of control Concentration Sleep Hearing Relaxation Quality of life emotion
TPFQ (28)	The primary way tinnitus impacts a person's life	20	0 completely disagree 100 completely agree	Grading system not provided. A higher total score indicates a higher level of tinnitus handicap	0.92 (28)	Concentration Emotion Hearing Sleep

* *The 40 out of 52 items are used for TQ total score, and the items 5 and 20 are counted double*.

The studies were used in which the outcomes of validated multi-item questionnaires were reported to measure the severity of depressive symptoms. These questionnaires are the Beck Depression Inventory (BDI), Hospital Anxiety and Depression Scale (HADS), Symptom Checklist Revised (SCL90-R) subscale depression, self-rating depression scale (SDS), the Center for Epidemiologic Studies Depression Scale (CES-D), Minnesota Multiphasic Personality Index (MMPI) subscale depression, and the Patient Health Questionnaire-9 (PHQ-9) (11, 12, 29–33). The definition of having a clinically relevant depressive symptom score was based on the provided cut-off score by the study reporting this outcome. If this definition or criterion was not provided, the cut-off score out of the manual or validation study describing the specific questionnaire was used to define “having a depression” (Table 1B).

**Table 1B:** Characteristics of12 depression/depressive symptom instruments.

**Instrument**	**Developed to measure:**	**Items**	**Scoring per item**	**Interpretation of score**	**Cronbach's α**	**Subscales**
Beck's Depression Inventory (BDI)-I (1961)/1A (1979) (34)	Cognitive, affective, somatic and vegetative symptoms of depression	21	0 not at all 1 mildly 2 moderately 3 severely	0-9 indicates minimal depression 10-18 indicates mild depression 19-29 indicates moderate depression 30-63 indicates severe depression	0.79-0.90 (35)	Cognitive-affective and Somatic-performance
BDI-II (1996) (11)	Cognitive, affective, somatic and vegetative symptoms of depression	21	0 not at all 1 mildly 2 moderately 3 severely	0-13 minimal range 14-19 mild depression 20-28 moderate depression 29-63 severe depression	0.93	Cognitive Affective Somatic
BDI- primary care (PC) / Fast-screen (FS) (2000)	Cognitive, affective, somatic and vegetative symptoms of depression	7	0 not at all 1 mildly 2 moderately 3 severely	0-3 minimal 4-8 mild depression 9-12 moderate depression 13-21 severe depression	0.85-0.89	Cognitive Affective
HADS	Anxiety and depression	7^*^	0-3 Likert scale	0-7 normal 8-10 mild 11-15 moderate 16+ severe	0.82 (36)	Anxiety Depression
SDS (30)	Depression severity	20	1 A little of the time 2 some of the time 3 good part of the time 4 most of the time	25-49 Normal 50-59 Mild to moderate 60-69 moderate to severe 70 and over Severe a score of 50 and higher suggests clinically significant symptoms.	0.68 (37)	none
Multiphasic Minnesota Personality Inventory (MMPI) short form (38)	Feelings of flow mood, lack of energy, suicidal ideation and other depressive features	26#	0 not at all 1 a little of the time 2 some of the time 3 a good deal of the time 4 almost all of the time	Very high scores may indicate depression, while moderate scores tend to reveal a general dissatisfaction with one's life.	0.53 (38)	Depression Psychasthenia Anxiety
PHQ-9 (33)	Depressed mood	9	0 Not at all 1 Several days a week 2 more than half the days 3 Nearly every day	≤ 4 minimal depression 5-9 mild depression 10-14 suggests moderate depression 15-19 moderately severe depression 20+ severe depression	0.86-0.89 (39)	none
CES-D (31)	Depressive symptoms and feelings	20	0 rarely or none of the time 1 Some or little of the time 2 moderately or much of the time 3 most or almost all the time	>16 suspect for a clinical depression	0.85 (40)	none
Symptom Checklist-90-revised (SCL90R) depression subscale (29)	Emotional distress	13^**^	0 Not at all 1 a little bit 2 Moderately 3 Quite a bit 4 Extremely	A higher score indicates more psychological distress	0.84 (41)	none

* *The 7 out of 14 questions regarding depression.*

** *The 13 out of 90 items regarding depression. #26 out of 370 questions regarding depression*.

Because of the expected heterogeneity of studies in methodology, inclusion criteria of participants, and assessed outcomes, the intentional analysis is a descriptive synthesis of results and not a meta-analysis.

## Results

### Search Strategy and Study Selection

The electronic search yielded a total of 2,298 articles. After removal of 386 duplicates, 1,912 studies were screened on title and abstract (see Figure 1). The title/abstract screening resulted in 183 studies to read in full text. A total of 150 studies were excluded due to various reasons; no correlation reported between tinnitus distress and depressive symptoms (*n* = 47); no tinnitus duration reported (*n* = 25); no validated TQ used (*n* = 29); no full text available (*n* = 23); only conference abstract (*n* = 7); descriptive articles without original data (*n* = 5); (systematic) reviews (*n* = 4); no use of a validated depression questionnaire (*n* = 4); pediatric studies (*n* = 2); letters to the editor (*n* = 1) or studies on acute tinnitus (*n* = 1). Finally, 33 studies (18.0%) were included in this review.

### Study Characteristics

Of the 33 included studies, 27 studies were cross-sectional cohort studies (16, 42–67) as discussed in the following: One was a retrospective cohort study (68), four were case–control studies (69–72), and one was a prospective study (73). The studies were published between 2000 and 2021 and described 8,990 participants (range: 44–1,416 participants per study) (Table 2).

**Table 2:** Study and patient characteristics.

**References, country**	**Study design**	***N* (m)**	**Age in years, (SD)**	**Population**	**Mean Tinnitus duration in years (SD)**	**Mean Hearing Loss [SD]**	**Used tinnitus questionnaire(s)**	**Used depression questionnaires**
Zachariae et al. (42), Denmark	CSC	50 (37)	22-79	Otorhinolaryngology outpatient clinic with complaints of tinnitus	5.2 (median 3.1)	NR	THI	BDI
Meric et al. (49), France	CSC	173(97)	49.6 (13.4)	NR	58.4(83) months	NR	TRQ	MMPI
Andersson et al. (71), Sweden	CCS	157(90) 86 (47)	46 (14) 51 (16)	Cohort recruited via the internet for a trail Otorhinolaryngology outpatient clinic with complaints of tinnitus	6.6 (7)	NR	TRQ	HADS-D
Monzani et al. (43), Italia	CSC	100 (63)	54.1 (14.4)	Otorhinolaryngology outpatient clinic with complaints of tinnitus	>3 months	53.7[14.9]*	THI	HADS-D
Oishi et al. (47), Japan	CSC	285(137)	59 (14)	Otorhinolaryngology outpatient clinic with complaints of tinnitus	4.3 (6.6)	37[22]⌢	THI	SDS
Ooms et al. (53), Belgium	CSC	139(87)	49 (13)	Otorhinolaryngology outpatient clinic with complaints of tinnitus	2.2(3.9)	37[22]**	THI	BDI
Milerova et al. (48), Germany	CSC	317(226)	49 (12.4)	Otorhinolaryngology and psychiatry outpatient clinics with complaints of tinnitus	>6 months	NR	THI, TRQ	SCL90R
Zirke et al. (44), Germany	CSC	100 (45)	49 (13)	Day ward for seven day multimodal tinnitus retraining therapy	>3 months	23.9[14.8]*	TQ	HADS-D
Granjeiro et al. (70), Brazil	CCS	68 (31)	36.8 (N.R.)	NR	5.95 (6.7)	NR***	THI	BDI
Zeman et al. (61), Germany	CSC	1,274 (N.R)	Median 52	Tinnitus Research Initiative database	5.0 (IQR 1.6; 11.9)	NR	THI	BDI
Oron et al. (62), Russia	CSC	50 (19)	49.8 (14.4)	Otorhinolaryngology outpatient clinic with complaints of tinnitus	49.8 (14.4)	NR	THI	BDI
Kleinstauber et al. (59), Germany	CSC	373 (217)	54.6 (12.1)	Internet based guided self-help group and cognitive-behavior group therapy group.	8.13 (7.66)	NR ♣	THI, Mini-TQ	HADS-D
Temugan eta l. (63), Turkey	CSC	44 (18)	37.84 (19.45)	Otorhinolaryngology outpatient clinic with complaints of tinnitus	17.8 months	NR ♦	THI	BDI
Trevis et al. (60), Australia	CSC	81 (34)	37.2 (7.0)	Community based sample via online advertisement	>3 months	NR 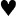	THI	BDI
Weidt et al. (64), Switzerland	CSC	208(135)	46.8 (14)	Otorhinolaryngology outpatient clinic with complaints of tinnitus	65.8 months (range 1-480)	NR	THI	BDI
Fackrell et al. (68), UK	RSC	294(212)	52.8 (N.R.)	Community based sample via online advertisement	9.0 (range 4 m-50 years)	Right: 19[13]~ Left 19[14]~	THI, THQ, TFI	BDI
Muller et al. (45), Sweden	CSC	260 (136)	62,4 Median	5th wave of the Swedish Longitudinal Occupational Survey of Health	NR	NR ♠	THI, TFI	HADS-D
Kehrle et al. (69), Brazil	CCS	84 (38)	37.2 (7.0)	Otorhinolaryngology outpatient clinic with complaints of tinnitus	5.6 (5.9)	NR***	THI	BDI
Hoff et al. (46), Sweden	CSC	100 (36)	51 (17)	Otorhinolaryngology outpatient clinic with complaints of tinnitus	>6 months (84%)	NR↔	THI, TFI	HADS-D
Wielopolski et al. (65), Switzerland	CSC	207 (133)	46.7 (13.9)	Otorhinolaryngology outpatient clinic with complaints of tinnitus	>1 month	NR	THI	BDI
Brueggemann et al. (73), Germany	PSC	311 (¬)	48.4 (11.6)	Participants of a seven day multimodal therapy of chronic tinnitus	>3 months	NR	TQ	CES-D
Han et al. (67), South Korea	CSC	248(114)	52.2 (13.4) male 55.8 (14.5) female	Otorhinolaryngology outpatient clinic with complaints of tinnitus	42.1 ± 81.2 29.1 ± 64.5	NRΨ NR Ψ	THI	BDI
Niemann et al. (51), Germany	CSC	1,416 (695)	49.8 (12.2)	Participants of a multimodal tinnitus-specific 7-day program (pre-treatment group)	5	NR	TQ	CES-D
Shin et al. (66), South Korea	CSC	79 (39)	53.1 (11.2)	Otorhinolaryngology outpatient clinic with complaints of tinnitus	3.5(3.4)	NR	THQ, TPFQ	BDI
Suzuki et al. (50), Japan	CSC	143 (85)	61 (24–85)	Otorhinolaryngology outpatient clinic with complaints of tinnitus	Male 42.1 (81.2) Female 29.1 (64.5)	NR NR	TFI, THI	SDS
Barozzi et al. (52), Italy	CSC	137 (83)	48.3 (14.1)	Otorhinolaryngology outpatient clinic with complaints of tinnitus	NR	NR	THI, TFI	BDI-PC
Boecking et al. (51), Germany	CSC	1,238 (614)	50.1 (12.0)	Self-referral to tinnitus outpatient clinic	NR (>3 months)	NR	TQ	CES-D
Fioretti et al. (54), Italy	CSC	107 (65)	49.1 (13.9)	Outpatient clinic with complaints of tinnitus	75.7% longer than 6 months.	NR⋎	TSCH, THI	BDI
Wang et al. (55), China	CSC	206 (106)	38.1 (12.4)	Patients visiting the outpatient clinic	0.8 (1.0)	NR	TFI	CES-D
Meijers et al. (56), Netherlands	CSC	308 (208)	51.8 (12.4)	Patients with chronic tinnitus visiting a tinnitus outpatient clinic	> 2 months	24.8[23.6]****	THI, TQ	SCL90R
Danioth et al. (72), Switzerland	CCS	75 (32)	52.3 (10)	Otorhinolaryngology outpatient clinic with complaints of tinnitus	9.8 (10.4)	NR	Mini-TQ	HADS-D
Huttenrauch et al. (57), Germany	CSC	316(127)	43.8 (14.4)	Online survey	10.2 (9.7)	NR	TFI	PHQ-9
Inagaki et al. (58), Japan	CSC	56(44)	57.0 (NR)	Outpatient clinic with complaints of tinnitus	Median 1.5 years	NR∞	THI	SDS

*m, male; CSC, Cross-sectional cohort; CCS, Case–control study, RCS, Retrospective Cohort Study, PCS, Prospective cohort study. ¬ = “equal proportions.” No exact data reported. NR, not reported. *mean PTA hearing threshold in Db (0,5-1-2-4 kHz) ** = mean PTA hearing threshold in Db (0,5-1-2 kHz) *** = only patients with “normal hearing” included. <25 dB hearing loss ⌢ = average pure tone hearing threshold in the ear experiencing tinnitus (500-1,000-2,000 Hz) ♣ = Hearing impairment: 24.7% unilateral, 48.5% bilateral. No hearing test preformed. ♦ = only hearing loss in patients with unilateral tinnitus reported (n=13). No mean score reported. 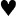 = 83% normal, 11% slight, 6% moderate hearing loss (n = 71) ~= mean PTA hearing threshold in Db (0,5-1-2-4-8 KHz) ♠ = 84.6% hearing problems. 15.4% no hearing problems. ↔= “subjects with varied degrees and types of hearing loss” Ψ= with hearing loss: male 45.6%, female 39.0% (no definition provided) ⋎= Based on the pure tone audiometry (250-8,000 Hz), 68 patients had a hearing loss. **** = Mean pure tone average hearing level (0,5, 1, 2, 4 kHz) of both ears. ∞ = presbycusis was present in 18 cases, sensorineural hearing loss in 11 cases, sudden deafness in 7 cases, and non-hearing loss tinnitus in 7 cases*.

### Risk of Bias Assessment

#### Quality of the Included Studies

The risk of bias assessment of included studies can be found in Supplementary Tables 1A,B. Four case–control studies were found. For these studies, the NOS assessment scale for case–control studies was used (69–72). All other studies were cohort studies for which the NOS assessment scale for cohort studies was used (16, 42–66, 73). Due to the study design the subsection “selection of the exposed cohort” and main sections “outcome,” and “comparability” were not achieved in these cohort studies. Because of the cross-sectional design of all cohort studies “demonstration that outcome of interest was not present at start of study” could not be judged.

#### Selection of Patients in the Cohort Studies

The representativeness of the exposed cohort was of poor quality in all included studies. In the 29 cohort studies, this was related to the highly selected patient groups included in those studies. The ascertainment of exposure was of good quality in these studies due to the use of the predefined TQs. The representativeness of the exposed cohort in the other four studies [Meric et al. (49), Shin et al. (66), Fioretti et al. (54), and Wang et al. (55)] was also judged to be of poor quality because of other reasons. Meric et al. (49) did not report about where the cohort was recruited from. Shin et al. (66), Fioretti et al. (54), and Wang et al. (55) gave no insight in the used selection procedure of the patients. The ascertainment of exposure was of good quality in all 29 studies due to the use of the predefined TQs.

#### Comparability of the Patients in the Cohort Studies

Among the cohort studies, only the study of Han et al. (67) compared two groups. Han et al. (67) compared the results of male and female patients with tinnitus. The other studies did not compare different study populations and therefore comparability was judged as not applicable.

#### Selection of Patients in the Case–Control Studies

The case definition was valid in all four case–control studies (Supplementary Table 1B). In the studies of Kehrle et al. (69), Granjeiro et al. (70), and Danioth et al. (72), the case definition was validated independently based on predefined inclusion criteria. Andersson et al. (71) recruited a cohort of patients with tinnitus from the internet and let them fill out questionnaires regarding their tinnitus distress and depressive symptoms. The representativeness was of low quality in all four case–control studies because of the potential selection bias. The method of selection of the control group was unclear in the studies of Kehrle et al. (69) and Granjeiro et al. (70). In the study of Andersson et al. (71), they recruited a control group from a clinical sample of patients which were seen by a psychologist because of tinnitus to compare to their internet cohort. Danioth recruited a control group using word of mouth advertisement. Only Kerle et al. (69) and Danioth et al. (72) had a control group with no history of tinnitus.

#### The Comparability and Expose of the Patients in Case–Control Studies

Among the four case–control studies, only Danioth et al. (72) and Kehrle et al. (69) controlled for multiple factors such as sex, age, and hearing thresholds. Granjeiro et al. (70), and Andersson et al. (71) did not provide the data regarding this subject. Exposure was scored of good quality in all studies.

### Data Extraction and Study Characteristics

#### Outcome Measures

The tinnitus distress was assessed by using different multi-item questionnaires as listed as follows: The THI was used in 20 out of 33 studies (42, 52–54, 56, 58, 60–66, 68–70, 74), TFI by seven (45, 46, 50, 52, 55, 57, 68), TQ by five (44, 48, 51, 56, 73), TRQ by two (49, 71), mini-TQ by two (59, 72), and both THQ (66) and TPFQ by one study (66). The depressive symptoms were also assessed by using different multi-item questionnaires. The BDI was used in 15 studies (42, 52–54, 60–63, 65, 66, 68, 69, 74), HADS-D was used by seven studies (43–46, 59, 71, 72), CES-D by four studies (16, 51, 55, 73), SDS by three studies (47, 50, 58), SCL-90R (48, 56). The MMPI and the PHQ-9 were both used once in two different studies (49, 57) (**Tables 4–6**).

### Outcomes

#### Studies Using BDI

The 15 out of 33 studies (45%) used the BDI to report depression severity (Table 3) (42, 52–54, 60–66, 68–70). Correlations between measures of tinnitus distress and depression severity were calculated using Pearson's test in 9 out of 15 studies (42, 52–54, 63, 65–68), Spearman's tests in four out of 15 studies (60, 62, 69, 70) and univariate regressions analysis in two of 15 studies (61, 64). The 13 out of 15 studies reported a statistical significant positive correlation, which varied between 0.51 and 0.73 in the studies using Spearman correlation and between 0.46 and 0.7 in the studies using Pearson's correlation (Table 3) (42, 52–54, 60–62, 64, 65, 67, 69, 70).

**Table 3:** Outcomes of studies using BDI to measure depressive symptoms/clinically relevant depressive symptom scores.

**Study, year**	**TQ**	**Tinnitus distress [mean (SD)]**	**Depressive symptoms questionnaire**	**Depressive symptom questionnaire score [mean (SD)]**	**Provided definition of clinically relevant depressive symptom scores**	**Prevalence of clinically relevant depressive symptom scores (%)**	**Correlation tinnitus distress and depressive symptoms (test used)**	**95% CI**	**Significance: *p***
Zachariae et al. (42)	THI	40.0 (22.3)	BDI-I	NR	NR	NR	0.73 (Pearson)	NR	<0.01
Granjeiro et al. (70)	THI	N.R.	BDI-I	NR	NR	33.8 (tinnitus) 13.0 (control)	0.506 (Spearman)	NR	<0.001
Zeman et al. (61)	THI	Median 46 IQR 30-66	BDI-I	9 (IQR 5:16)	According to BDI manual**	NR.	R^2^ 0.46 B 0.26∞	NR	<0.001
Oron et al. (62)	THI	N.R.	BDI-I	NR	NR	NR	0.725 (Spearman)	NR	0,01
Wielopolski et al. (65)	THI	44.4 (23.3)	BDI-I	9.3 (6.9)	*****	11.6	0.70 (Pearson)	NR	<0.001
Shin et al. (66)	THQ TPFQ	34.5 (20.6) 40.8 (23.6)	BDI-I	14.5 (12.5)	NR	NR	N.R. 0.55 (Pearson)	NR NR	N.R. <0.01
Han et al. (67)	THI male THI female	38.3 (25.9) 43.0 (5.9)	BDI-I BDI-I	8.7 (9.8) 11.6 (9.3)	******	NR NR	0.606 (Pearson) 0.395(Pearson)	NR NR	<0.01 <0.01
Ooms et al. (53)	THI	44.12 (22.72)	BDI-II	11.27 (9.45)	According to BDI manual**	5.9	0.38 (Pearson)	NR	<0.01
Temugan et al. (63)	THI	37.84 (19.45)*	BDI-II÷	8.95 (6.78)*	***	NR	0.271 (Pearson)	NR	0.82
Trevis et al. (60)	THI	26.32 (95% 21.79–30.85)	BDI-II	9.9.5 (7.62–12.28)	According to BDI manual**	6	0.51 (Spearman)	NR	<0.001
Weidt et al. (64)	THI	44.3 (23)	BDI-II	9.2 (7)	BDI ≥ 18 points	11.5	0.50 (R^2^), B=0.21	NR	<0.001
Fackrell et al. (68)	THI THQ TFI	37.6 (20.1) 41.3 (17.9) 40.6 (20.1)	BDI-II	8.4 (8.2)	NR	NR	0.60 (Pearson) 0.53 (Pearson) 0.57 (Pearson)	NR	N.R.
Kehrle et al. (69)	THI	N.R.	BDI-II	NR	****	41.6	0.651 (Spearman)	NR	<0.001
Fioretti et al. (54)	TSCH, THI	M 39.0 (25.4) F 43.7 (21.8)	BDI-II	M 9.1 (6.6) F 12.5 (8.3)	According to BDI**	NR	0.68 (Pearson)	NR	<0.001
Barozzi et al. (52)	THI, TFI	NR	BDI-PC	NR	NR	NR	0.46 (pearson)	NR	<0.01

Abbreviations and symbols:. ÷, referred to in text as Becks depression scale; NR, not reported; THI, Tinnitus Handicap Inventory; TFI, Tinnitus Functional Index; TQ, tinnitus Questionnaire; THQ, Tinnitus Handicap Questionnaire; TPFQ, Tinnitus Primary Function Questionnaire; TSCH, Tinnitus Sample Case History Questionnaire; ^*^, Discrepancies were found in the THI score and BDI score comparing the data out of the text to the data out of the tables of the included studies. This table present the scores presented in the article's table. In the text scores are: THI 38.04 (20.03), BDI 9.46 (6.49); ∞, a linear regression was used; ^**^, BDI-I: 0–9 minimal, 10–18 mild, 19–29 moderate, 39–63 severe. / BDI-II: 0–13 minimal , 14–19 mild, 20–28 moderate, 29–63 Severe; ^***^, 1–10 normal 11–16 slight psychological breakdown 1–20 borderline clinic depression 21–30 moderate depression 31–40 serious depression >40 very high depression; ^****^, 0–11 minimal 12–19 mild 20–35 moderate 36–63 severe; ^*****^, 0–10 corresponds with no or minimal depression, of 11–17 with a mild or moderate depression, of 18–63 with a clinical relevant depression; ^******^, 0–9 no depression, 10–15 mild depression, 16–23 moderate depression 24-63 severe depression.

All 15 studies except Shin et al. (66) used the THI to measure tinnitus distress. Fackrell et al. (68) described the correlation between BDI and THI; THQ and TFI using a Pearson correlation (THI 0.60, THQ 0.53, TFI 0.57) without reporting an estimate or significance. Shin et al. (66) found a significant correlation using the TPFQ questionnaire [*r* = 0.55 *p* < 0.01 using Pearson correlation, confidence interval (CI) which was not reported], and they did not report on the correlation with THQ scores.

#### Studies Using Hospital Anxiety and Depression Scale

The 7 out of 33 studies (23%) studies used the HADS-D to report depressive symptom severity (43–46, 59, 71, 72) (Table 4). Both Hoff et al. (46) and Andersson et al. (71) found significant positive correlations between tinnitus distress and severity of depression using a Pearson correlation (Hoff: *r* = 0.6, no *p*-value reported, CI = 0.43–0.70; Andersson: *r* = 0.54 *p* < 0.001 no CI value reported). Monzani et al. (43) also used a Pearson correlation and found a positive significant relation but referred to this as “a significant emotional distress increase” instead of a depression. Four other studies reported significant positive correlations ranging between 0.32–0.526 for studies using Spearman's correlation (44, 45, 59, 71). These correlations were found in studies in which the TQ, TRQ, and/or TFI were used (Zirke: TQ: 0.32, no CI value reported, *p* < 0.001; Muller: THI: 0.42, no CI value reported, *p* < 0.01, TFI: 40 no CI value reported, *p* < 0.01; Kleinstauber: THI 0.579, no CI value reported, *p* < 0.001).

**Table 4:** Outcomes of studies using HADS to measure depressive symptoms/clinically relevant depressive symptom scores.

**Study, year**	**TQ**	**Tinnitus distress [mean (SD)]**	**Depressive symptoms questionnaire**	**Depressive symptom questionnaire score [mean (SD)]**	**Provided definition of clinically relevant depressive symptom scores**	**Prevalence of clinically relevant depressive symptom scores (%)**	**Correlation tinnitus distress and depressive symptoms (test used)**	**95% CI**	**Significance: *p***
Andersson et al. (71)	TRQ	Internet 38.2 (22.0) outpatient clinic 27.7(21.1)	HADS-D HADS-D	6.5 (3.9) 5.1 (4.1)	HADS > 11 points	17 15	0.69 (Pearson) 0.54 (Pearson)	NR NR	<0.001 <0.001
Monzani et al. (43)	THI	27.2 (19.8)	HADS-D	9.5 (4.1)	HADS > 8 points *	NR.	0.693 (Pearson)	NR	0.01
Zirke et al. (44)	TQ	35.0 (16.1)	HADS-D	5.1 (3.3)	NR	NR	0.32 (Spearman)	NR	0.001
Kleinstauber et al. (59)	THI mTQ	41.33 (18.46) 13.01 (4.74)	HADS-D	5.91 (4.0)	NR	NR	0.579 (Spearman) NR	NR	<0.001
Muller et al. (45)	THI TFI	Median 24 (IQR 14 - 38) median 24 (IQR 14 - 38)	HADS-D	4.0 (IQR 2 – 8)	NR	4.6*	0.42 (Spearman) 0.40	NR	<0.01 <0.01
Hoff et al. (46)	THI TFI	41.7 (21.3) 55.3 (19.9)	HADS-D	5.0 (3.5)	NR	NR	NR 0.60 (Pearson)	0.43-0.73	N.R.
Danioth et al. (72)	mTQ	10.6 (7.1)	HADS-D	5.3(3.7)	NR	NR	0.526 (Spearman)	NR	<0.01

Abbreviations and symbols: ^*^, defined as: ‘abnormal high score; NR, not reported; THI, Tinnitus Handicap Inventory; TFI, Tinnitus Functional Index; TQ, Tinnitus Questionnaire; TRQ, Tinnitus Reaction Questionnaire; mTQ, Mini Tinnitus Questionnaire; HADS-D; Hospital Anxiety and Depression Scale; ^*^; Values over 10, for anxiety, and over 8 for depression, correspond to a significant emotional distress increase.

#### Studies Using Other Scales Than BDI or HADS

The 11 of 33 studies (33%) used another instrument to measure depressive symptoms rather than BDI or HADS-D (16, 47–51, 55–58, 73) (Table 5).

**Table 5:** Outcomes of studies using other questionnaires to measure depressive symptoms/clinically relevant depressive symptom scores.

**Study, year**	**TQ**	**Tinnitus distress [mean (SD)]**	**Depressive symptoms questionnaire**	**Depressive symptom questionnaire score [mean (SD)]**	**Provided definition of clinically relevant depressive symptom scores**	**Prevalence of clinically relevant depressive symptom scores (%)**	**Correlation tinnitus distress and depressive symptoms (test used)**	**95% CI**	**Significance: *p***
Oishi, 2011(43)	THI	56 (24)	SDS	44 (10)	+1 SD above the average score	39	F = 20.7 *	NR	<0.01
Suzuki et al. (50)	TFI THI	NR NR	SDS	NR NR	NR	NR NR	0.548 (Pearson) NR	NR	<0.01 NR
Inagaki et al. (58)	THI	55.3 (27)	SDS	44.4 (10.5)	‘a higher SDS score indicates more severe depressive symptoms'	NR	0.539 (Pearson)	NR	<0.01
Niemann et al. (51)	TQ	38.3 (17.1)	CES-D	18.0 (11.6)	NR	N.R.	0.630 (Spearman)	NR	<0.01
Brueggemann et al. (73)	TQ	37.9 (16.6)	CES-D	18.2 (11.6)	>23 points	N.R.	0.493 (Pearson)	NR	<0.01
Boecking et al. (16)	TQ	39.5 (17.0)	CES-D	18.3 (11.9)	>23 major depressive disorder.	NR	1.31 (Cohen's)	1.17–1.44	<0.05
Wang et al. (55)	TFI	33.1 (22.8)	CES-D (*n* = 161)	NR	Score >20	NR	0.334 (Pearson)	NR	<0.01
Milerova et al. (48)	THI TQ	N.R.	SCL-90R- sub depression	N.R	NR	N.R	0.504 (Pearson) 0.425 (Pearson)	NR	<0.01 <0.01
Meijers et al. (56)	THI TQ	45.6 (22.9) 39.6 (16.7)	SCL-90R sub depression	26.4	NR	17.3	B= 1.56 ** B= 0.97**	1.50–1.62 0.92–1.02	<0.01 <0.01
Meric et al. (49)	TRQ	34.65 (24.94)	MMPI sub depression (abbreviated version)	NA	+2 SD from mean	12	r= 0.44 (Pearson)	NR	<0.01
Huttenrauch et al. (57)	TFI	40.0 (23.5)	PHQ-9	NR	NR	NR	0.56 (Spearman)	NR	<0.001

Abbreviations and symbols: THI, Tinnitus Handicap Inventory; TFI, Tinnitus Functional Index; TQ, Tinnitus Questionnaire; TRQ, Tinnitus Reaction Questionnaire; NR, Not Reported; M, male; F, Female; SDS, Self-rating depression scale (Zung, 1965); CES-D, Center of Epidemiologic studies Depression scale; SCL90R, Symptom Checklist-90-revised; MMPI, Multiphasic Minnesota Personality Inventory; PHQ, Patient Health Questionnaire; ^**^, a linear regression was used; ^*^, multiple linear regression with THI as an independent variable.

Oishi et al. (47) used the SDS and found a statistical significant positive correlation (*F* = 20.7, *p* < 0.01, no CI value reported) with the THI using a multiple linear regression. Suzuki et al. (50) and Inagaki et al. (58) also used the SDS and both found a statistical significant positive correlations with the THI using a Pearson correlation (Suzuki: 0.548, *p* < 0.01, no CI value reported, Inagaki: 0.539, *p* > 0.01, no CI value reported).

Milerová et al. (48) and Meijers et al. (56) used the subscale depression of the SCL-90R questionnaire and both found a statistical significant positive correlation with THI and TQ using different statistical tests (Milerova—SCL90-THI: 0.504 (Pearson) *p* < 0.01, no CI value reported, SCL-TQ: 0.425 (Pearson), *p* < 0.01, no CI reported; Meijers—SCL90-THI: *B* = 1.56 (linear regression), *p* < 0.01. CI = 1.50–1.92, SCL90-TQ: 0.97 (linear regression), *p* < 0.01, CI = 0.92–1.02).

Meric et al. (49) used the subscale depression of a French adaptation of the MMPI and found a statistical significant positive correlation with the TRQ using a Pearson correlation (0.44, *p* < 0.01 no CI value reported).

Niemann et al. (51), Brueggemann et al. (73) and Boecking et al. (16) used the CES-D and found statistical significant positive results using the TQ [Niemann: 0.63 (Spearman) *p* < 0.01, no CI value reported; Brueggeman: 0.493 (Pearson), *p* < 0.01, no CI value reported; Boecking: 1.31 (Cohen's), CI: 1.17–1.44, *p* < 0.01]. Wang et al. (55) used the CES-D questionnaire in combination with the TFI and reported a significantly positive score of 0.334 using a Pearson correlation (*p* < 0.01, no CI value reported).

#### Data Regarding Prevalence of Clinically Relevant Depressive Symptom Scores

Among the 33 studies, 11 studies reported on the prevalence of clinical relevant depressive symptom scores (45, 47, 49, 53, 56, 60, 64, 65, 69–71). Of these studies, in six studies, this was based on the BDI; in two studies, this was based on HADS outcomes; and in three studies, this was based on other questionnaires. The reported prevalence range varied from 4.6–41.6% in these 11 studies.

## Discussion

In this systematic review, we investigated the correlation between the experienced tinnitus distress and the severity of depressive symptoms in patients with chronic tinnitus using validated questionnaires. Also, the prevalence of clinically relevant depressive symptoms or a clinical depression in patients with chronic tinnitus was evaluated. We systematically searched three databases using predefined inclusion and exclusion criteria. A total of 33 studies were included with a total of 8,990 patients with chronic tinnitus of which 29 were cohort studies and four case-control studies, all with a high risk of bias. All included studies reported a significant positive correlation between tinnitus distress and the severity of depressive symptoms, mostly with moderate to strong correlations.

Multiple factors could have influenced the results of this review. First, included studies were judged as having a high risk of bias and described highly selected patient groups. This high risk of bias was caused by vague inclusion and exclusion criteria, relatively small patient groups and the use of a cross-sectional study design in all but four studies. In addition, in the majority of the included studies patients were investigated that were recruited in outpatient (tinnitus) clinics or by using online advertisements, which makes these studies more prone for selection bias. Patients selected by the fact that they seek medical help for their tinnitus or react on online advertisements may differ from others with tinnitus and from those who never seek any medical help (75). Furthermore, only 4 out of the 33 included studies reported on the hearing status of the study participants. Hearing loss in itself is associated with a higher risk of depression and also is common in patients with chronic tinnitus (76). Therefore, the lack of information about hearing loss as a confounder of the association between tinnitus and depression is regarded as an important limitation of the outcomes of the current study.

Most of the included studies used either the BDI or HADS-D questionnaire to measure levels of depressive symptoms in patients with chronic tinnitus but only 11 studies reported on the prevalence of clinical relevant depressive symptom scores (45, 47, 49, 56, 60, 64, 65, 69–71, 74). The cut-off scores of a “significant” depression were not defined uniformly in the selected studies. For example, in the 15 studies who used the BDI, only 4 studies referred to the original BDI manual for interpretation of the score (53, 54, 60, 61). Some studies provided different interpretations of scores using the same measure (63, 65, 67, 69) and most studies did not provide a definition at all of a cut-off for a clinical relevant depressive symptom scores (42, 52, 62, 66, 68, 69). The wide variety of questionnaires, definitions and cut-off values used to measure and define (symptoms of) depression could all contribute to the wide range of reported prevalence numbers of clinically relevant depressive symptom scores (4.6–41.7%).

When the mean depression scores of the included studies are interpreted based on the questionnaire specific scoring system, the overall minimal-to-mild BDI depression scores were found. In addition, the reported mean HADS-D scores of the included studies should be interpreted as “normal” and mean CES-D scores were far below the definition of a clinical depression/severe depressive symptoms. This could indicate that even if there is a statistically significant positive correlation between tinnitus distress and severity of depressive symptoms, a clinical symptomatic depression was relatively rare in studied patients with chronic tinnitus. This is of special interest as most studies were describing participants recruited in outpatient clinics or by using online advertisements of which it is more likely that participants were having a high tinnitus impact and/or in need for medical help.

The questionnaire design itself could also have influenced the results. Ooms et al. (53) reported about overlap in the content of frequently used depressive symptom questionnaires and TQs. When comparing THI to the BDI questionnaire, the similarities in the content of 15 out of the 25 questions of the THI with 13 out of 21 questions of the BDI were found. This was partly explained by the similarities between the symptoms mentioned by patients with tinnitus or with a depression (e.g., insomnia, concentration difficulties, annoyance, and fear) (77). The THI, SCL90-R, and HADS-D also demonstrated an overlap in questions, but in lesser effect. This is potentially caused by the design of HADS-D, were questions of somatic symptoms are excluded from the questionnaire (36). Beside this, also the differences in TQ design itself could have influenced the results. Because different TQs are designed to measure different aspects of tinnitus, their exposure to psychological facets of tinnitus differs. For example, the TRQ consists for 77% out of questionnaires which investigate the psychological/emotional effects of tinnitus, whereas the THQ only does so for 44% (78). Possibly, this results in stronger correlations between tinnitus and depression in more psychological aimed questionnaires. This could be an important factor to explain the findings of our study with overall significant positive correlations found between tinnitus distress and the severity of depressive symptoms. This raises questions about the validity to distinguish symptoms of both entities. On the other hand, this also underlines the close relationship between both disorders and warrants a multidisciplinary diagnostic and therapeutically approach to treat individuals based on their needs.

## Conclusions

All of the included studies found statistically significant correlations between the experienced tinnitus distress and the severity of depressive symptoms in patients with chronic tinnitus. The reported prevalence of having a clinically relevant depressive symptom scores varied from 4.6–41.6% in included studies, but mean depression scores were low in all studies. This contrast, in combination with the quality of studies and risk of bias makes it impossible to conclude about the stated relationship based on current evidence. Besides this, the heterogeneity in used tinnitus and depression symptoms scores and the overlap between the content of these questionnaires hinders drawing conclusions. To answer this question, the future population-based studies with updated methodology will be necessary.

## Data Availability Statement

The original contributions presented in the study are included in the article/Supplementary Material, further inquiries can be directed to the corresponding author/s.

## Author Contributions

SM, RM, and MR contributed to articles screening. SM, MR, AS, and IS were involved in critical appraisal. All authors revised the manuscript and contributed to the interpretation of the results and approved the final version of this study.

## Conflict of Interest

The authors declare that the research was conducted in the absence of any commercial or financial relationships that could be construed as a potential conflict of interest.

## Publisher's Note

All claims expressed in this article are solely those of the authors and do not necessarily represent those of their affiliated organizations, or those of the publisher, the editors and the reviewers. Any product that may be evaluated in this article, or claim that may be made by its manufacturer, is not guaranteed or endorsed by the publisher.
