# Supplementary material for: Correlation Between Chronic Tinnitus Distress and Symptoms of Depression: A Systematic Review
Source: Front Neurol. 2022 May 2;13:870433. doi: 10.3389/fneur.2022.870433 (PMC9108431; doi:10.3389/fneur.2022.870433)
Supplement: Supplementary file 1 [file Table_1.docx]

**Search strategy:**

**Pubmed:**
((((((tinnitus[MeSH Terms]) OR tinni*[Title/Abstract]) OR THI[Title/Abstract]) OR Tinnitus handicap inventory[Title/Abstract]) OR TQ[Title/Abstract]) OR Tinnitus questionnaire[Title/Abstract]) OR Tinnitus severity[Title/Abstract] [N=13090]

**AND**

(((((((((((((((depression[MeSH Terms]) OR depressive disorder[MeSH Terms]) OR depres*[Title/Abstract]) OR symptom checklist[Title/Abstract]) OR SCL-90*[Title/Abstract]) OR short form health survey[Title/Abstract]) OR SF-36[Title/Abstract]) OR Coping inventory for stressful situation[Title/Abstract]) OR CISS[Title/Abstract]) OR Hospital anxiety depression scale[Title/Abstract]) OR HADS[Title/Abstract]) OR Becks depression scale[Title/Abstract]) OR BDS[Title/Abstract]) OR Becks depression inventory[Title/Abstract]) OR BDI[Title/Abstract]) OR Hamilton[Title/Abstract] [N=445901]

**1 AND 2 = 802**

**Embase:**
'tinnitus'/exp OR 'tinni*':ab,ti OR 'thi':ab,ti OR 'tinnitus handicap inventory':ab,ti OR 'tq':ab,ti OR 'tinnitus questionnaire':ab,ti OR 'tinnitus severity':ab,ti [N=21905]

AND

'depression'/exp/mj OR 'depres*':ab,ti OR 'symptom checklist':ab,ti OR 'scl-90*':ab,ti OR 'short form health survey':ab,ti OR 'sf-36':ab,ti OR 'coping inventory for stressful situation':ab,ti OR 'ciss':ab,ti OR 'hospital anxiety depression scale':ab,ti OR 'hads':ab,ti OR 'becks depression scale':ab,ti OR 'bds':ab,ti OR 'becks depression inventory':ab,ti OR 'bdi':ab,ti OR 'hamilton depression rating scale':ab,ti [N=576468]

**1 AND 2 = 1197**

**Cochrane:**

Tinnitus or THI or tinnitus Handicap inventory or TQ or Tinnitus Questionnaire or tinnitus severity:ti,ab,kw (Word variations have been searched) [N=1757]

AND

depression or depressive disorder or symptom checklist or SCL-90 or short form health survey or SF-36 or coping inventory for stressful situation or CISS or hospital anxiety depression scale or HADS or becks depression scale or DBS or becks depression inventory or BDI or hamilton:ti,ab,kw (Word variations have been searched)
[N=54040]

**1 AND 2 = 287**
