# Supplementary material for: Correlation Between Chronic Tinnitus Distress and Symptoms of Depression: A Systematic Review
Source: Front Neurol. 2022 May 2;13:870433. doi: 10.3389/fneur.2022.870433 (PMC9108431; doi:10.3389/fneur.2022.870433)
Supplement: Supplementary file 2 [file Data_Sheet_1.docx]

**Supplementary Material**

**Legend Newcastle –Ottawa quality assessment scale for cohort studies:**

**Selection**

1) Representativeness of the exposed cohort
a) Truly representative ***(one star)***
b) Somewhat representative ***(one star)***
c) Selected group
d) No description of the derivation of the cohort

**2) Selection of the non-exposed cohort**
a) Drawn from the same community as the exposed cohort ***(one star)***
b) Drawn from a different source
c) No description of the derivation of the non-exposed cohort

**3) Ascertainment of exposure**
a) Secure record (e.g., surgical record) ***(one star)***
b) Structured interview ***(one star)***
c) Written self-report
d) No description
e) Other

**4) Demonstration that outcome of interest was not present at start of study**
a) Yes ***(one star)***
b) No

**Comparability**

1) Comparability of cohorts on the basis of the design or analysis controlled for confounders
a) The study controls for age, sex and marital status ***(one star)***
b) Study controls for other factors (list) _________________________________ ***(one star)***
c) Cohorts are not comparable on the basis of the design or analysis controlled for confounders

**Outcome**

1) Assessment of outcome
a) Independent blind assessment ***(one star)***
b) Record linkage ***(one star)***
c) Self report
d) No description
e) Other

**2) Was follow-up long enough for outcomes to occur**
a) Yes ***(one star)***
b) No

Indicate the median duration of follow-up and a brief rationale for the assessment above:____________________

**3) Adequacy of follow-up of cohorts**
a) Complete follow up- all subject accounted for ***(one star)***
b) Subjects lost to follow up unlikely to introduce bias- number lost less than or equal to 20% or description of those lost suggested no different from those followed. ***(one star)***
c) Follow up rate less than 80% and no description of those lost
d) No statement

**Good quality:** 3 or 4

stars in selection domain AND 1 or 2 stars in comparability domain AND 2 or 3 stars in outcome/exposure domain

**Fair quality:** 2 stars in selection domain AND 1 or 2 stars in comparability domain AND 2 or 3 stars in outcome/exposure domain

**Poor quality:** 0 or 1 star in selection domain OR 0 stars in comparability domain OR 0 or 1 stars in outcome/exposure domain.

**Legend Newcastle- Ottawa Quality assessment scale for case control studies**

**Selection:**

1) the case definition adequate?

a) yes, with independent validation ***(one star)***

b) yes, eg record linkage or based on self-reports

c) no description

2) Representativeness of the cases

a) consecutive or obviously representative series of cases ***(one star)***

b) potential for selection biases or not stated

3) Selection of Controls

a) community controls ***(one star)***

b) hospital controls

c) no description

4) Definition of Controls

a) no history of disease (endpoint) ***(one star)***

b) no description of source

**Comparability:**1) Comparability of cases and controls on the basis of the design or analysis

a) study controls for _______________ (Select the most important factor.) ***(one star)***

b) study controls for any additional factor ***(one star)*** (This criteria could be modified to indicate specific control for a second important factor.)

**Exposure:**

1) Ascertainment of exposure

a) secure record (eg surgical records) ***(one star)***

b) structured interview where blind to case/control status ***(one star)***

c) interview not blinded to case/control status

d) written self-report or medical record only

e) no description

2) Same method of ascertainment for cases and controls

a) yes ***(one star)***

b) no

3) Non-Response rate

a) same rate for both groups ***(one star)***

b) non respondents described

c) rate different and no designation
